# Genetic diversity of *Mycobacterium tuberculosis* isolates from northwest of Iran during COVID-19 era

**DOI:** 10.1186/s43042-023-00383-4

**Published:** 2023-01-09

**Authors:** Peyvand Kashi, Farzaneh Pakdel, Mohammad Hossein Soroush Barhaghi, Mohammad Ahangarzadeh Rezaee, Sepehr Taghizadeh, Javid Sadeghi, Mehdi Yousefi, Reza Ghotaslou, Mohammad Asgharzadeh, Pourya Gholizadeh, Hossein Samadi Kafil

**Affiliations:** 1 Student Research Committee, Tabriz University of Medical Sciences, Tabriz, Iran; 2 Drug Applied Research Center, Tabriz University of Medical Sciences, Tabriz, Iran; 3 Immunology Research Center, Tabriz University of Medical Sciences, Tabriz, Iran; 4 Biotechnology Research Center, Tabriz University of Medical Sciences, Tabriz, Iran; 5 Research Center for Pharmaceutical Nanotechnology, Tabriz University of Medical Sciences, Tabriz, Iran

**Keywords:** *Mycobacterium tuberculosis*, MIRU-VNTR, Epidemiology, Genotyping, COVID-19

## Abstract

**Background:**

Tuberculosis (TB) is considered one of the most infectious diseases in the world. In this study, we intended to examine the epidemiology of tuberculosis by MIRU-VNTR to define the changes that occur in the transmission of tuberculosis in the region during the COVID-19 era. A total of 120 *Mycobacterium tuberculosis* isolates were collected from sputum samples of patients referred to East Azerbaijan Center TB from December 2020 to August 2021. Demographic information such as age, sex, place of birth, previous TB history, and relevant medical data was collected. The proportion method was performed for drug susceptibility testing, and the PCR-based MIRU-VNTR method was applied to identify molecular epidemiology relationships.

**Results:**

The isolates were collected from 78 male (65%) and 39 female (32.5%) Iranian patients and 3 (2.5%) Azerbaijani patients. Ninety-three distinct patterns were identified including 15 clustered patterns and 36 unique patterns. The largest cluster was composed of seven isolates. Furthermore, one cluster with 5 members, four clusters with 3 members, and nine clusters with 2 members. In MIRU-VNTR typing, 75 clusters belonged to the Tabriz region and just 3 to the Republic of Azerbaijan. All isolates were sensitive to rifampin, isoniazid, and ethambutol.

**Conclusions:**

Results of the current study showed COVID-19 pandemic had a direct effect on the transmission and diagnosis of tuberculosis. Less diagnosis and less clustering can indicate public controls and hygiene, and the use of masks had a direct effect on the transmission and diagnosis of tuberculosis. However, misidentification and less focus on other respiratory infections are expected during the pandemic. Studies on the co-infection of COVID-19 and tuberculosis and the role of mask and sanitization against TB are strongly recommended.

## Introduction

Tuberculosis (TB) is considered one of the most infectious diseases worldwide. This disease is commonly caused by different species of *Mycobacterium tuberculosis* (MTB). Recently, the World Health Organization (WHO) reported tuberculosis as one of the consistent infections in 2021 and TB remained the most common cause of death from a single bacterial infectious pathogen with 1.6 million patients died from TB [1, 2]. Globally, an estimated 10 million people have developed TB infection and there were an estimated 1.2 million deaths every year [3, 4]. Thirty countries also reported the emergence of new resistant cases, with India leading the way, followed by China, Indonesia, the Philippines, Pakistan, Nigeria, Bangladesh, and South Africa [5]. Despite widespread Bacillus Calmette–Guerin (BCG) vaccination and anti-tuberculosis drugs, tuberculosis remained its place as the leading cause of bacterial infection-based death [6]. The emergence of the coronavirus disease of 2019 (COVID-19) during 2019 was a new era for human beings with about 5 million deaths and more than 100 million infected people by the end of 2021 [7–9]. It had a direct effect on different life aspects, even the epidemiology of other infectious diseases. Besides health problems, more restrictions and higher sanitary protocols had an impact on the epidemic of other infectious diseases, especially respiratory diseases [10, 11]. TB is considered as a risk factor for COVID-19, and patients with TB should be prioritized for COVID-19 therapy and prevention strategies [12].

Mycobacterial Interspersed Repetitive Units-Variable Number of Tandem Repeats (MIRU-VNTR) is a polymerase chain reaction (PCR)-based typing method, which determines the size and repeated number of units in each locus by amplifying the mycobacterial interspersed repetitive units [13]. Easy operation, economical cost, reproducible results, and high discriminatory power make it practical for routine use, and the digital results from this method can be compared and exchanged easily between different laboratories [14]. MIRU typing relies on the variation in copy number of tandem repeat (VNTR) loci, and it simply needs basic PCR and electrophoresis apparatus. TB isolation wards have been re-purposed to manage COVID-19 patients, while TB/infectious disease specialists are being redirected to COVID-19 care [15]. Molecular typing of the MTB complex is a powerful tool to examine the dispersion of tubercle bacilli in outbreaks and to analyze the transmission of tuberculosis as well as to distinguish between recent TB infection and reactivation of latent infection [16, 17]. Tuberculosis can infect all parts of the body, but the most common form is pulmonary tuberculosis which in adults is usually accompanied by a positive sputum smear and is highly contagious [18]. Tuberculosis is pulmonary in 85% of cases and extrapulmonary in 15% of cases [19]. TB and COVID-19 affect the same site (organ) and studying their impact on each other can be interesting. Therefore, in this study, we evaluated the molecular epidemiology of tuberculosis during the COVID-19 era. Due to the nature of tuberculosis infection over the long period, our study was based on the transmission of tuberculosis in positive cases despite their infection with COVID-19, and only the transmission pattern of tuberculosis was studied.

## Methods

### Sample collection and processing

A total of 120 *M. tuberculosis* isolates were collected from sputum samples of patients referred to East Azerbaijan Center TB from December 2020 to August 2021. Demographic information such as age, sex, place of birth, previous TB history, and relevant medical data was collected by the statistics of the tuberculosis center, and then recorded in corresponding forms. This study was approved by the local ethical committee (IR.TBZMED.REC.1399.146). Primary isolation and culture from sputum samples were performed in accordance with standard protocols.

### Drug susceptibility testing

The drug susceptibility testing was performed by the proportion method as described before [13]. A bacterial suspension was prepared according to the 0.5 McFarland turbidity standards and was diluted 1:10. 0.2 ml of the suspension was added to Löwenstein–Jensen medium (Thermo Fisher Scientific Inc., USA) without antibiotics and Löwenstein–Jensen medium containing individual antibiotics including 40 μg/ml rifampin, 0.2 μg/ml isoniazid, and 2 μg/ml ethambutol (Sigma-Aldrich, Missouri, USA). The mediums were incubated at 37 °C for up to 6 weeks, and antibiotic resistance was considered as if the number of mycobacterial colonies at a concentration of each antibiotic was more than 1% compared to medium without antibiotics [20].

### Genomic DNA extraction

Genomic DNA was extracted from inactivated cultured bacteria, by a simple boiling method. We conducted DNA extraction by the phenol–chloroform method as previously described [21]. All extracted DNAs were stored at − 70 °C for further analysis. DNA of the isolates was extracted using the cetyltrimethylammonium bromide (CTAB, Sigma-Aldrich, USA) phenol–chloroform method [22]. In this regard, 15 selected loci were amplified by the primer-specific PCR method. All reactions were done in 20 µL volumes using the Takara master mix. The thermal protocol included 94 °C for 7 min, followed by 35 cycles of 94 °C for 45 s, and different temperatures for annealing at 72 °C for 55 s. In this study, we used the multiplex PCR method. The annealing temperatures were as follows: MIRU 2 (65 °C), MIRU 4 and MIRU 31 (63 °C), MIRU 10 and MIRU 24 (60 °C), MIRU 16 and MIRU 26 (65 °C), MIRU 20 (59 °C), MIRU 23 and MIRU 39 (67 °C), MIRU 27 and MIRU 40 (64 °C), ETR-A (66 °C), ETR-B (68 °C) and ETR-C (69 °C). Electrophoresis of PCR products was also performed on 1.5% agarose gel and stained with ethidium bromide [23]. All sizes of fragments were determined by a 50 bp DNA ladder size marker.

### MIRU-VNTR typing

MIRU-VNTR typing was performed by PCR analysis with specific primers based on standard 15 loci defined in Supply et al. [24]. Patients with similar numeric MIRU-VNTR results were considered as the same cluster of transmission. Patterns of MIRU-VNTR refer to numeric results of MIRU-VNTR typing based on 15 loci of VNTR. PCR products were analyzed by electrophoresis on 1.5% agarose gels using a 100 bp DNA ladder as size markers. The obtained size was compared by applying online apparatus at (http://www.MIRU-VNTRplus.org). The dendrogram was obtained using the unweighted pair group method with arithmetic mean (UPGMA) algorithm analysis. Other statistical comparisons were made by chi-square test, and a *P* value below 0.05 was considered significant.

## Results

### Study population

The study involved 120 *M. tuberculosis*-positive cultures, obtained from new cases of pulmonary TB in northwestern provinces of Iran for 9 months from December 2020 to August 2021. Of these, 78 males (65%) and 39 females (32.5%) were Iranians, and 3 (2.5%) Azerbaijani patients. The age mean of patients was 62 years with a range from 2 to 90 years. The Republic of Azerbaijani patients constitute 2 males and 1 female; the age range was 35–62 years. All isolates were susceptible to rifampin, isoniazid, and ethambutol.

### MIRU-VNTR typing

**Fig. 1:**
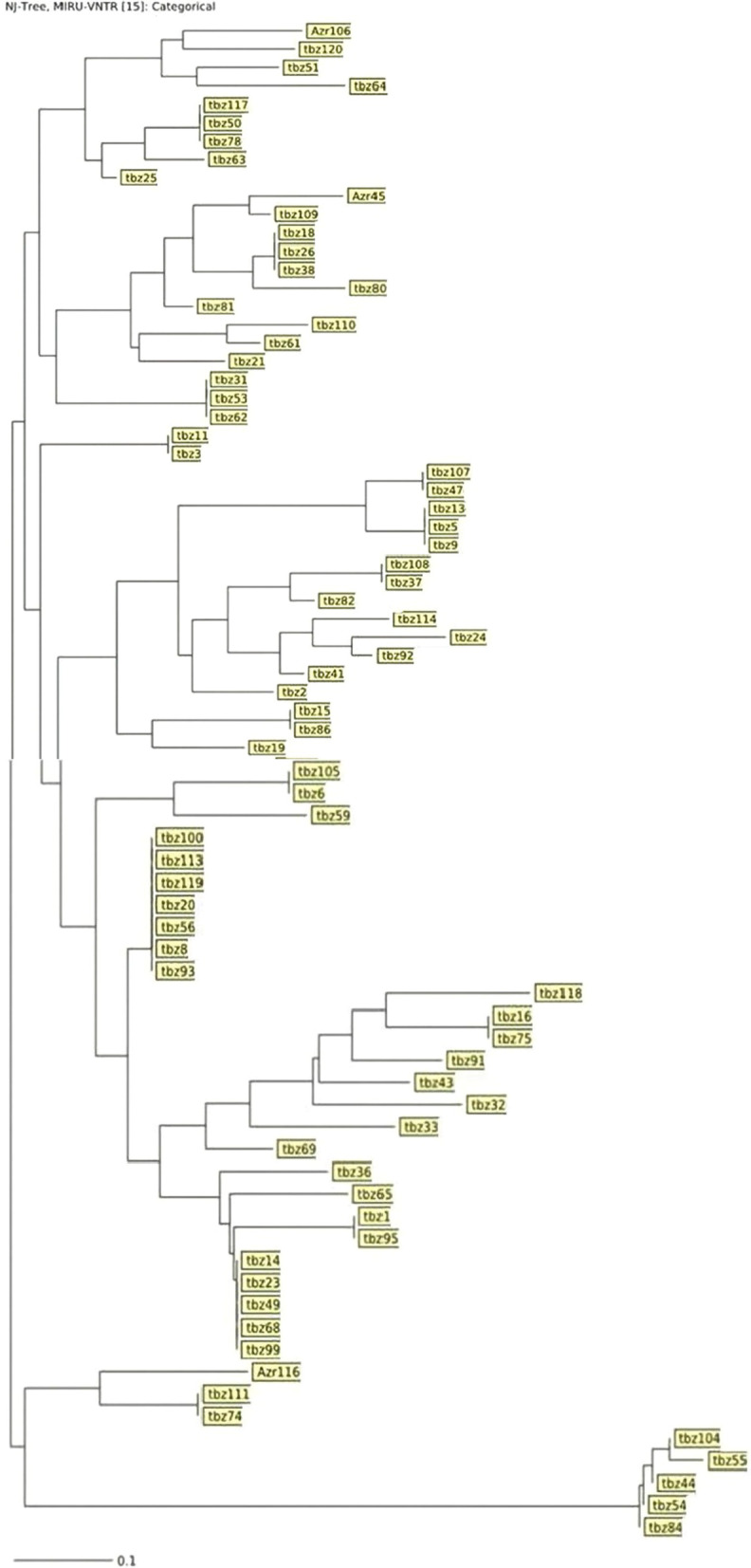
Dendrogram of MIRU-VNTR pattern of 120 *M. tuberculosis* isolates obtained from new TB cases in Tabriz. This dendrogram shows clusters and samples with similar cluster based on MIRU-VNTR typing in 15 loci. The biggest cluster consists of 7 patients from Tabriz. Patients from Tabriz were identified by name started with tbz and patients from Azerbaijan were identified by names started with Azr. Number indicates random numbers assigned to each patient

Using MIRU-VNTR, 120 distinct patterns were identified including 15 clustered patterns and 36 unique patterns. The largest cluster was composed of seven isolates. Furthermore, one cluster with 5 members, 4 clusters with 3 members, and 9 clusters with 2 members. In MIRU-VNTR typing, 75 clusters belonged to the Tabriz region and just 3 to the Republic of Azerbaijan (Fig. 1).

### Molecular epidemiology analysis

**Fig. 2:**
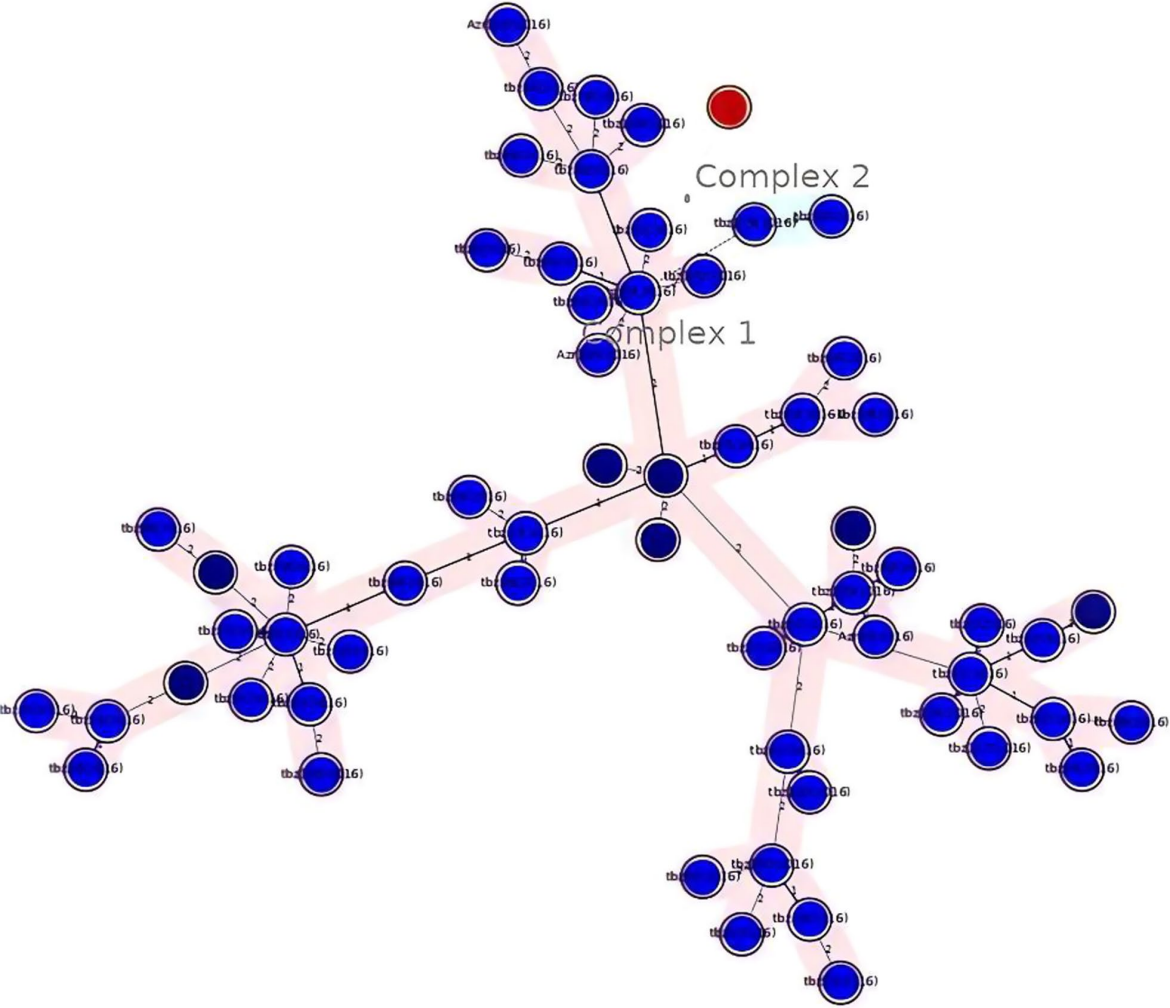
Relationship of the MIRU-VNTR pattern of non-cluster isolates with other categories. The blue circle indicates the MIRU-VNTR pattern isolates and the dark circles indicate the number of isolates in a cluster. This figure shows how diverse are clusters from each other and they not too close to each other to consider as the same cluster by reducing loci numbers

Genetic relationships between isolates using both weighted and non-weighted group methods by algorithm (NJ) method of MIRU and ETR data were inferred. MIRU-VNTRplus software for statistical and genetic analysis containing UPGMA and NJ trees, which provides the calculation of allelic diversity and relationships with other reference species, was used, and the distances between the strains were calculated according to the number of VNTR copies, and the UPGMA phylogenetic tree was drawn by the mentioned software (Figs. 1 and 2).

## Discussion

All isolates of *M. tuberculosis* were identified by conventional methods, and their molecular relationships were investigated by MIRU-VNTR typing. According to the results, we had reduced clonal transmission of *M. tuberculosis* during this pandemic. In our study, the system based on 15 loci was used, which is the most widely used among the different sets of MIRU-VNTR loci [24]. This study was the first to study during the COVID-19 pandemic. We aimed to determine the dynamics of tuberculosis transmission in the region. Our samples were collected from the Central TB laboratory in Tabriz, and DNA was extracted after the inactivation of the pathogens. In the past few years, due to the influx of migrants to the East Azerbaijan region, the incidence of tuberculosis has increased, but in the last 2 years, due to the widespread outbreak of COVID-19 and quarantine in different countries, migration to this region and transmission of tuberculosis have decreased sharply. Our study shows that the rate of person-to-person transmission has decreased in comparison with previous studies in this region. Most of the isolates that were previously isolated have been placed in fewer clusters in similar studies in the past in the northwestern region of Iran. In this study, we had 15 clusters with 32.5% of the patients included in the clusters and the biggest cluster was composed of 7 isolates and only two clusters had more than 3 members. During 2005–2007, a study by a similar method in the region showed 38% of isolates included in the clusters and 21 clusters of transmission revealed by the MIRU-VNTR method [13]. In a similar study exactly before the current study, 194 isolates from the same regions were examined by the MIRU-VNTR method, 25 clusters were identified with an 84% clustering rate and the biggest cluster included 33 members [25]. Statistical comparison on clustering shows no significant difference between our study and Asgharzadeh (2011) (*P* = 0.36), but significantly different with clustering observed by Mahdavipoor (2018) (*P* < 0.001) [13, 25]. These data show an increasing rate of clustering and transmission of tuberculosis right before the current study. In the current study, we had fewer patients from the Republic of Azerbaijan with only 6 patients from this country. However, all of these patients were included in the clusters, which shows how important is the role of these patients in the transmission of tuberculosis. This increase was due to the COVID-19 pandemic and restrictions on people crossing borders and therefore Azerbaijani patients had fewer roles in TB transmission in the region. By comparing results from the current study, despite increasing TB diagnosis and transmission before COVID-19, several factors caused less transmission and diagnosis of TB infection in this region. These factors can be due to fewer people trips, increased use of sanitizers and hygiene, quarantine in the regions and border restrictions, fewer uses of public transport systems, fewer handshakes, use of masks, less gathering, parties, and maybe less diagnosis because of more focus on COVID-19 diagnosis and treatment. Limitations of this study were less participation of hospitals to send their tuberculosis samples to the TB center, due to high waves of COVID-19 and problems due to COVID-19 pandemics such as restrictions, laboratories concentration on COVID-19 detection, and less material availability.

In conclusion, the results of the current study showed that, during the COVID-19 pandemic, less transmission of tuberculosis occurred from the Republic of Azerbaijan patients and fewer clusters were defined among patients. The COVID-19 pandemic may affect the transmission and diagnosis of tuberculosis. Less diagnosis and less clustering may indicate public controls and hygiene, and the use of masks can be one of the direct effects on the transmission and diagnosis of tuberculosis. However, misidentification and less focus on other respiratory infections are expected during pandemics. Studies on co-infection of COVID-19 and tuberculosis and the role of mask and sanitization against TB are strongly recommended.

## Data Availability

The data supporting this research project are available from the corresponding author upon request.

## References

[1] WHO (2022) Tuberculosis deaths and disease increase during the COVID-19 pandemic. WHO (World Health Organisation). https://www.who.int/news/item/27-10-2022-tuberculosis-deaths-and-disease-increase-during-the-covid-19-pandemic. Accessed 27 Oct 2022

[2] Fathizadeh H, Hayat SMG, Dao S, Ganbarov K, Tanomand A, Asgharzadeh M, Kafil HS (2020) Long non-coding RNA molecules in tuberculosis. Int J Biol Macromol 156:340–346 32283111 10.1016/j.ijbiomac.2020.04.030

[3] Organization WH (2020) Global tuberculosis report 2020: executive summary

[4] Leylabadlo HE, Kafil HS, Yousefi M, Aghazadeh M, Asgharzadeh M (2016) Pulmonary tuberculosis diagnosis: Where we are? Tuberc Respir Dis 79(3):134–142 10.4046/trd.2016.79.3.134 PMC4943897 27433173

[5] Pourostadi M, Rashedi J, Poor BM, Kafil HS, Shirazi S, Asgharzadeh M (2016) Molecular diversity of *Mycobacterium tuberculosis* strains in Northwestern Iran. Jundishapur J Microbiol 9(9) 10.5812/jjm.35520 PMC5086081 27800145

[6] WHO (2022) Global tuberculosis report 2022. World Health Organisation

[7] Gholizadeh P, Safari R, Marofi P, Zeinalzadeh E, Pagliano P, Ganbarov K, Esposito S, Khodadadi E, Yousefi M, Kafil HS (2020) Alteration of liver biomarkers in patients with SARS-CoV-2 (COVID-19). J Inflamm Res 13:285 32669866 10.2147/JIR.S257078 PMC7335895

[8] Gholizadeh P, Sanogo M, Oumarou A, Mohamed MN, Cissoko Y, Sow MS, Pagliano P, Akouda P, Soufiane SA, Iknane AA, Oury M, Diallo S, Köse Ş, Dao S, Kafil HS (2021) Fighting COVID-19 in West Africa after experiencing the Ebola epidemic. Health Promot Perspect 11(1):5 33758750 10.34172/hpp.2021.02 PMC7967127

[9] Khodadadi E, Maroufi P, Khodadadi E, Esposito I, Ganbarov K, Espsoito S, Yousefi M, Zeinalzadeh E, Kafil HS (2020) Study of combining virtual screening and antiviral treatments of the Sars-CoV-2 (Covid-19). Microb Pathog 145:104241 10.1016/j.micpath.2020.104241 PMC7199731 32387389

[10] Fathizadeh H, Taghizadeh S, Safari R, Khiabani SS, Babak B, Hamzavi F, Ganbarov K, Esposito S, Zeinalzadeh E, Dao S, Köse Ş, Kafil HS (2020) Study presence of COVID-19 (SARS-CoV-2) in the sweat of patients infected with COVID-19. Microb Pathog 149:104556–104556. 10.1016/j.micpath.2020.104556 33031898 10.1016/j.micpath.2020.104556 PMC7534876

[11] Ozma MA, Maroufi P, Khodadadi E, Köse Ş, Esposito I, Ganbarov K, Dao S, Esposito S, Dal T, Zeinalzadeh E (2020) Clinical manifestation, diagnosis, prevention and control of SARS-CoV-2 (COVID-19) during the outbreak period. Infez Med 28(2):153–165 32275257

[12] Nicolas Casco ea (2022) Tuberculosis and COVID-19 co-infection: description of the global cohort. Eur Respir J 59(3):2102538. 10.1183/13993003.02538-2021 10.1183/13993003.02538-2021 PMC8588566 34764184

[13] Asgharzadeh M, Kafil HS, Roudsary AA, Hanifi GR (2011) Tuberculosis transmission in Northwest of Iran: using MIRU-VNTR, ETR-VNTR and IS6110-RFLP methods. Infect Genet Evol 11(1):124–131 20951237 10.1016/j.meegid.2010.09.013

[14] Asgharzadeh M, Khakpour M, Salehi TZ, Kafil HS (2007) Use of mycobacterial interspersed repetitive unit-variable-number tandem repeat typing to study *Mycobacterium tuberculosis* isolates from East Azarbaijan province of Iran. Pak J Biol Sci 10(21):3769–3777 19090229 10.3923/pjbs.2007.3769.3777

[15] Malik AA, Safdar N, Chandir S, Khan U, Khowaja S, Riaz N, Maniar R, Jaswal M, Khan AJ, Hussain H (2020) Tuberculosis control and care in the era of COVID-19. Health Policy Plan 35(8):1130–1132 32832996 10.1093/heapol/czaa109 PMC7499582

[16] Mathema B, Kurepina NE, Bifani PJ, Kreiswirth BN (2006) Molecular epidemiology of tuberculosis: current insights. Clin Microbiol Rev 19(4):658–685 17041139 10.1128/CMR.00061-05 PMC1592690

[17] Narayanan S (2004) Molecular epidemiology of tuberculosis. Indian J Med Res 120:233–247 15520480

[18] Bennett JE, Dolin R, Blaser MJ (2014) Mandell, douglas, and bennett’s principles and practice of infectious diseases: 2-volume set, vol 2. Elsevier Health Sciences, Edinburgh

[19] Arsang S, Kazemnejad A, Amani F (2011) Epidemiology of tuberculosis in Iran (2001–08). J Gorgan Univ Med Sci 13(3):78–86

[20] Bento CM, Gomes MS, Silva T (2021) Evolution of antibacterial drug screening methods: current prospects for mycobacteria. Microorganisms 9(12):2562 34946162 10.3390/microorganisms9122562 PMC8708102

[21] Asgharzadeh M, Shahbabian K, Samadi Kafil H, Rafi A (2007) Use of DNA fingerprinting in identifying the source case of tubercolosis in East Azarbaijan province of Iran. J Med Sci 7(3):418–421

[22] Asgharzadeh M, Mazloumi A, Kafil HS, Ghazanchaei A (2007) Mannose-binding lectin gene and promoter polymorphism in visceral leishmaniasis caused by *Leishmania infantum*. Pak J Biol Sci 10(11):1850–1854 19086549 10.3923/pjbs.2007.1850.1854

[23] Kafil HS, Mobarez AM (2015) Assessment of biofilm formation by enterococci isolates from urinary tract infections with different virulence profiles. J King Saud Univ-Sci 27(4):312–317

[24] Supply P, Mazars E, Lesjean S, Vincent V, Gicquel B, Locht C (2000) Variable human minisatellite-like regions in the *Mycobacterium tuberculosis* genome. Mol Microbiol 36(3):762–771 10844663 10.1046/j.1365-2958.2000.01905.x

[25] Mahdavipoor B, Asgharzadeh M, Hajibonabi F, Rashedi J, Pourostadi M, Bahador TN, Asadi N, Kafil HS, Barhaghi MHS (2018) Genotyping of *Mycobacterium tuberculosis* isolates from northwest Iran for determination on the mechanism of transmission. Trop Biomed 35(3):619–626 33601749

